# Prevalence and correlates of depressive symptoms and competitive anxiety in tennis players: a cross-sectional study

**DOI:** 10.3389/fpubh.2026.1920866

**Published:** 2026-08-26

**Authors:** Marc Niering, Emilia Richter, Julius Riedel, Rainer Beurskens, Johanna Seifert

**Affiliations:** 1 Department of Psychiatry, Social Psychiatry, and Psychotherapy, Hannover Medical School, Hannover, Germany; 2 Institute of Biomechanics and Neurosciences, Nordic Science, Hannover, Germany; 3 Triagon Academy Munich, School of Sports, Psychology and Education, Ismaning, Germany; 4 Department of Sports Medicine and Health Promotion, Friedrich-Schiller-University Jena, Jena, Germany; 5 Department of Health and Social Affairs, FHM Bielefeld - University of Applied Sciences, Bielefeld, Germany

**Keywords:** athlete mental health, competitive anxiety, depressive symptoms, sport psychiatry, sport psychology

## Abstract

**Background:**

Tennis is a psychologically demanding individual sport characterized by high competitive pressure and self-regulatory demands. However, data on depressive symptoms and competitive anxiety in tennis players remain limited.

**Objectives:**

To assess the prevalence of depressive symptoms and competitive anxiety in active tennis players and identify demographic and sport-related correlates.

**Methods:**

In this cross-sectional online survey, 313 active tennis players (mean age 26.9 ± 10.2 years) completed the Beck Depression Inventory-II (BDI-II) and the Sport Competition Anxiety Test (SCAT). Associations between psychological outcomes and demographic and sport-related variables were examined using correlation and regression analyses.

**Results:**

Clinically relevant depressive symptoms were reported by 33.5% of participants. Female athletes reported higher competitive anxiety than male athletes (*p* = 0.003). Competitive anxiety was positively associated with depressive symptoms (*r* = 0.119, *p* = 0.036). A previous diagnosis of depression was the strongest predictor of clinically relevant depressive symptoms (*OR* = 6.81, 95% *CI* [2.22, 20.91]). Previous depression diagnosis, recent injury or illness, and psychotropic medication use were associated with greater depressive symptom severity.

**Conclusion:**

Competitive tennis players demonstrated a substantial burden of depressive symptoms and competitive anxiety, with one third of athletes reporting clinically relevant depressive symptoms. Competitive anxiety was associated with depressive symptoms, indicating overlapping aspects of psychological distress. Athletes with a history of depression, recent injury or illness, or psychotropic medication use may represent groups with an increased burden of depressive symptoms and may benefit from closer monitoring. Routine mental health screening, integrated psychological support, and targeted interventions during injury rehabilitation should be considered to promote athlete well-being and reduce persistent psychological distress.

## Introduction

Recognition of mental health symptoms in athletes has increased substantially within sports medicine and sports psychiatry. Symptoms of anxiety and depression occur frequently in competitive and high-performance athletes, although reported prevalence estimates vary considerably across athletic populations and study designs (1–3).

Recent systematic reviews further suggest that depressive symptoms may affect up to one third of competitive athletes, with higher symptom burden reported particularly among female athletes and athletes competing in individual sports (4, 5). This psychological burden has additionally been associated with athlete-specific stressors such as injury, career instability, public scrutiny, and performance-related pressure (3, 6). Athletes competing in individual sports may experience greater personal responsibility for performance outcomes and fewer opportunities for interpersonal support during competition. Tennis places particularly high demands on self-regulation, as athletes must repeatedly manage their emotions, attention, and tactical decisions throughout a match with limited opportunities for external coaching. Additional sport-specific stressors include prolonged travel periods, ranking-based performance evaluation, financial uncertainty outside the highest competitive levels, and sustained competitive exposure across the season (7). While elevated levels of competitive anxiety have been reported in tennis players, particularly among female athletes (8, 9), data specifically addressing depressive symptoms in tennis populations remain limited. Considering the frequent co-occurrence of anxiety and depressive symptoms in athletes (2, 6, 10), their relationship in tennis players remains insufficiently understood. In addition, injury burden and prior mental health problems have previously been associated with psychological distress in athletes (3, 11), but have rarely been examined in competitive tennis populations.

Our study therefore investigated depressive symptoms and competitive anxiety in active tennis players, focusing on prevalence, co-occurrence, and demographic as well as sport-related correlates.

## Methods

### Procedures

This cross-sectional online survey was conducted and reported in accordance with the Strengthening the Reporting of Observational Studies in Epidemiology (STROBE) guidelines (12). The survey was completed anonymously and provided in both German and English. Participants from North America completed the English questionnaire versions, whereas participants from Germany predominantly completed the German versions, with three participants choosing the English version. Eligibility criteria were defined *a priori* and applied consistently across all participants.

Data collection was conducted between February 8, 2024, and February 16, 2025 using an online survey platform. The survey link was distributed through tennis associations, clubs, and individual athletes in German-speaking European countries and North America using a convenience sampling approach to obtain a heterogeneous sample. The questionnaire assessed demographic characteristics, tennis background, depressive symptoms, and competitive anxiety.

To be eligible for participation, individuals had to be classified as Tier 2 (Trained/Developmental) or Tier 3 (Highly Trained/National Level) athletes according to the Participant Classification Framework (13). Eligibility further required a minimum of 3 years of organized training and competitive experience, active participation in regular tennis training or competition, and tennis as the primary competitive sport.

The study was conducted in accordance with the Declaration of Helsinki and approved by the local ethics committee of the Nordic Science Institute of Biomechanics and Neurosciences, Hannover, Germany. All participants provided informed consent prior to participation.

### Participants

A total of 313 participants were included in the analysis (Table 1). An *a priori* power analysis was conducted using G*Power (version 3.1; Heinrich-Heine-Universität Düsseldorf, Germany) for the planned multiple regression analyses. Assuming a small-to-moderate effect size (*f^2^* = 0.05), an alpha level of 0.05, a statistical power of 0.80, and eight predictors, the required minimum sample size was estimated at 263 participants (14). The final sample therefore exceeded the required sample size for the planned analyses.

**Table 1:** Characteristics of the tennis players surveyed (*n* = 313).

Variable	*n*	% of participants	M ± SD
Age (years)	–	–	26.91 ± 10.23
Tennis experience (years)	–	–	17.29 ± 7.74
Sex			
Female	156	49.8	–
Male	156	49.8	–
Non-binary	1	0.3	–
Education			
None	16	5.1	–
High school	121	38.7	–
Vocational school*	9	2.9	–
Bachelor’s	107	34.2	–
Master’s	60	19.2	–
Employment status			
Student	113	36.1	–
Working part-time	58	18.5	–
Working full-time	136	43.5	–
Retired	2	0.6	–
Unable to work	4	1.3	–
Health-related variables			
Diagnosed depression (past)	20	6.4	–
Suicide attempt (past)	9	2.9	–
Taking psychotropic medication	39	12.5	–
Current location			
Europe	239	76.4	–
North America	74	23.6	–

*A German dual education system which combines practical training in a business with theoretical education in a vocational school (German: “Berufsschule”).

Of the included participants, 156 identified as male, 156 as female, and one as non-binary. Participants self-reported their sex using the response options male, female, and non-binary. The mean age was 26.91 ± 10.23 years, and the athletes reported an average tennis experience of 17.29 ± 7.74 years. Twenty participants reported a previous diagnosis of depression, nine reported a history of suicide attempt, and 39 reported current psychotropic medication use. At the time of participation, 239 athletes were located in Europe and 74 in North America.

Participants additionally reported whether they had experienced a serious injury or illness within the previous 6 months. No further information regarding injury type, illness type, severity, time-loss duration, current status, or whether the condition was tennis-related was collected.

### Instruments

Symptoms of depression were assessed using the Beck Depression Inventory-II (BDI-II) (15), a widely used 21-item self-report instrument assessing depressive symptom severity. The questionnaire evaluates cognitive, emotional, and somatic aspects of depression, including sadness, pessimism, guilt, sleep disturbance, and physical discomfort. Items are rated on a four-point scale, with higher total scores indicating greater depressive symptom severity.

According to established cut-off values, scores of 0–13 indicate minimal depressive symptoms, 14–19 mild depressive symptoms, 20–28 moderate depressive symptoms, and 29–63 severe depressive symptoms. Previous studies have demonstrated good internal consistency and reliability of the BDI-II across different populations (16).

Competition anxiety was assessed using the Sport Competition Anxiety Test (SCAT) (17), a 15-item self-report instrument measuring athletes’ general tendency to experience anxiety during competitive situations. Items are rated on a three-point scale ranging from 1 (“rarely”) to 3 (“often”), with higher total scores reflecting greater competitive trait anxiety. SCAT scores below 17 indicate low competitive anxiety, scores between 17 and 24 moderate anxiety, and scores above 24 high competitive anxiety (18). The SCAT has demonstrated good psychometric properties, including high internal consistency and acceptable test–retest reliability in previous validation studies (17, 19). In the present sample, internal consistency was good for the BDI-II (Cronbach’s *α* = 0.886) and acceptable for the SCAT (Cronbach’s α = 0.667).

### Statistical analysis

Statistical analyses were performed using JASP software [Version 0.19.3; (20)]. Depressive symptoms and competitive anxiety represented the primary psychological outcomes.

Descriptive statistics, including means (*M*), standard deviations (*SD*), frequencies, and percentages, were calculated to summarize demographic characteristics and questionnaire scores. Independent-samples *t* tests were conducted to examine group differences in BDI-II and SCAT scores across selected demographic and health-related variables. Cohen’s *d* was calculated as an effect size for all independent-samples *t* tests. Values of 0.2, 0.5, and 0.8 were interpreted as small, moderate, and large effects, respectively. Pearson correlation coefficients (*r*) were calculated to examine associations between demographic, clinical, and psychological variables and the outcome measures.

Exploratory moderation analyses were conducted to examine whether the association between competitive anxiety and depressive symptoms differed according to sex, previous diagnosis of depression, and serious injury or illness within the previous 6 months. Separate linear regression models were estimated with BDI-II scores as the dependent variable and SCAT scores, the respective moderator, and the SCAT × moderator interaction term as predictors. Significant interaction effects were interpreted as evidence of moderation.

A multivariable logistic regression analysis was performed to identify predictors of clinically relevant depressive symptoms, with BDI-II scores ≥ 14 coded as 1 and scores < 14 coded as 0. In addition, multivariable linear regression analyses were conducted for BDI-II and SCAT scores to examine predictors of symptom severity. The following variables were included as potential predictors: tennis experience, previous diagnosis of depression, previous suicide attempt, serious injury or illness within the previous 6 months, psychotropic medication use, age, sex, and current location.

Dichotomous variables were coded as follows: female = 0 and male = 1; no = 0 and yes = 1; Europe = 0 and North America = 1. The non-binary participant was included in all descriptive analyses but excluded from regression analyses involving sex because meaningful estimation was not possible for a single observation. For logistic regression models, odds ratios (*ORs*), 95% confidence intervals (*CIs*), and Nagelkerke’s *R^2^* are reported. For linear regression and moderation models, standardized beta coefficients (*β*), *p* values, and adjusted *R^2^* values are presented. For the linear regression models, assumptions of multicollinearity, homoscedasticity, residual distributions, and influential observations were evaluated. For the logistic regression model, multicollinearity and influential observations were assessed, and model fit was evaluated using Nagelkerke’s *R^2^*. Statistical significance was set at *p* < 0.05. Only complete cases were included in the respective analyses, and no imputation procedures were applied.

## Results

### Prevalence and severity of self-reported depressive symptoms and competitive anxiety

Prevalence rates and severity distributions for depressive symptoms and competitive anxiety are presented in Table 2. Overall, 33.5% (95% CI: 28.5–38.9%; *n* = 105) of the athletes screened positive for clinically relevant depressive symptoms (BDI-II ≥ 14). Among these athletes, the mean BDI-II score was *M* = 21.9 (*SD* = 8.7), corresponding to moderate depressive symptom severity. Mild depressive symptoms were observed in 16.9% (*n* = 53) of the athletes, moderate symptoms in 12.5% (*n* = 39), and severe symptoms in 4.2% (*n* = 13).

**Table 2:** Prevalence and severity of depressive symptoms and competitive anxiety among athletes.

BDI-II severity (cut-off scores)	*n*	Mean (SD)	% of *n*
Minimal (not clinically relevant; 0–13)	208	5.4 (4.3)	66.5
Clinically relevant depressive symptoms (14–63)	105	21.9 (8.7)	33.5
Mild (14–19)	53	16.2 (1.4)	16.9
Moderate (20–28)	39	23.0 (3.0)	12.5
Severe (29–63)	13	41.7 (6.7)	4.2
SCAT severity (cut-off-scores)			
Low anxiety (<17)	59	14.2 (2.6)	18.8
Moderate anxiety (17–24)	160	20.4 (2.1)	51.1
High anxiety (>24)	94	27.5 (1.9)	30.0

*n*, number of participants; BDI-II, Beck Depression Inventory-II; SCAT, Sport Competition Anxiety Test; SD, standard deviation.

Regarding competitive anxiety, 18.8% (*n* = 59) of the participants demonstrated low anxiety levels, whereas 51.1% (*n* = 160) reported moderate anxiety and 30.0% (*n* = 94) demonstrated high competitive anxiety levels.

### Group differences in depressive symptoms and competitive anxiety

**Figure 1:**
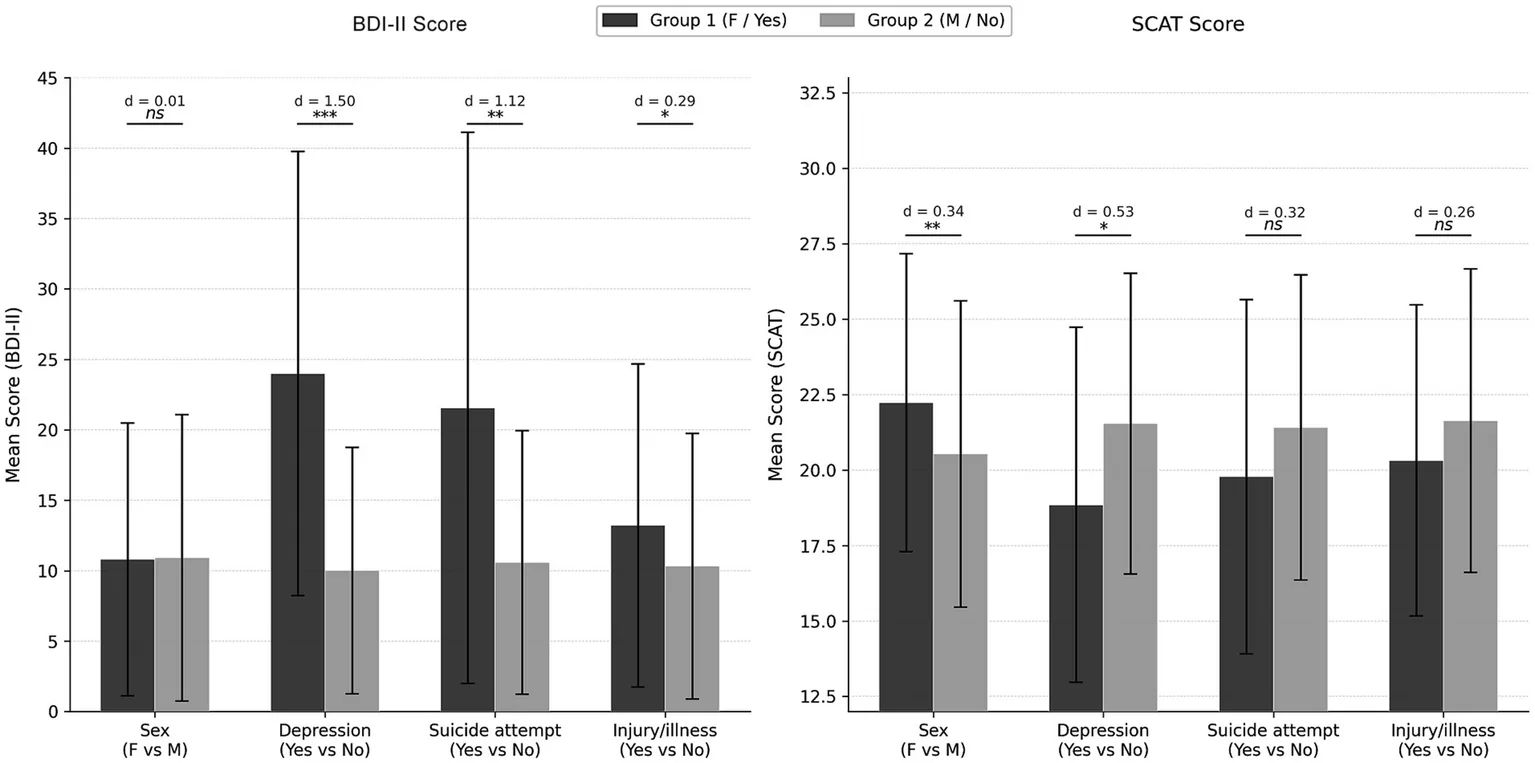
Group differences in depressive symptoms and competitive anxiety across selected health-related variables. Bars represent group means, and error bars indicate standard deviations.

Figure 1 illustrates group differences in depressive symptoms and competitive anxiety across selected health-related variables, with full results provided in Supplementary Table S1. Female athletes reported significantly higher competitive anxiety scores than male athletes, *t*(310) = 3.00, *p* = 0.003, *d* = 0.34. No significant sex differences were observed for depressive symptom severity.

Athletes with a previous diagnosis of depression reported significantly higher BDI-II scores than athletes without a previous diagnosis, *t*(311) = −6.49, *p* < 0.001, *d* = 1.50. In contrast, these athletes reported significantly lower competitive anxiety scores, *t*(311) = 2.30, *p* = 0.022, *d* = 0.53.

Similarly, athletes reporting a previous suicide attempt showed significantly higher depressive symptom severity, *t*(311) = −3.32, *p* = 0.001, *d* = 1.12, whereas no significant differences were observed for competitive anxiety. Athletes reporting a serious injury or illness within the previous 6 months additionally demonstrated significantly higher BDI-II scores than athletes without recent injury or illness, *t*(311) = −2.07, *p* = 0.039, *d* = 0.29. No significant group differences in competitive anxiety were observed for recent injury or illness.

### Correlates of depressive symptoms and competitive anxiety

**Figure 2:**
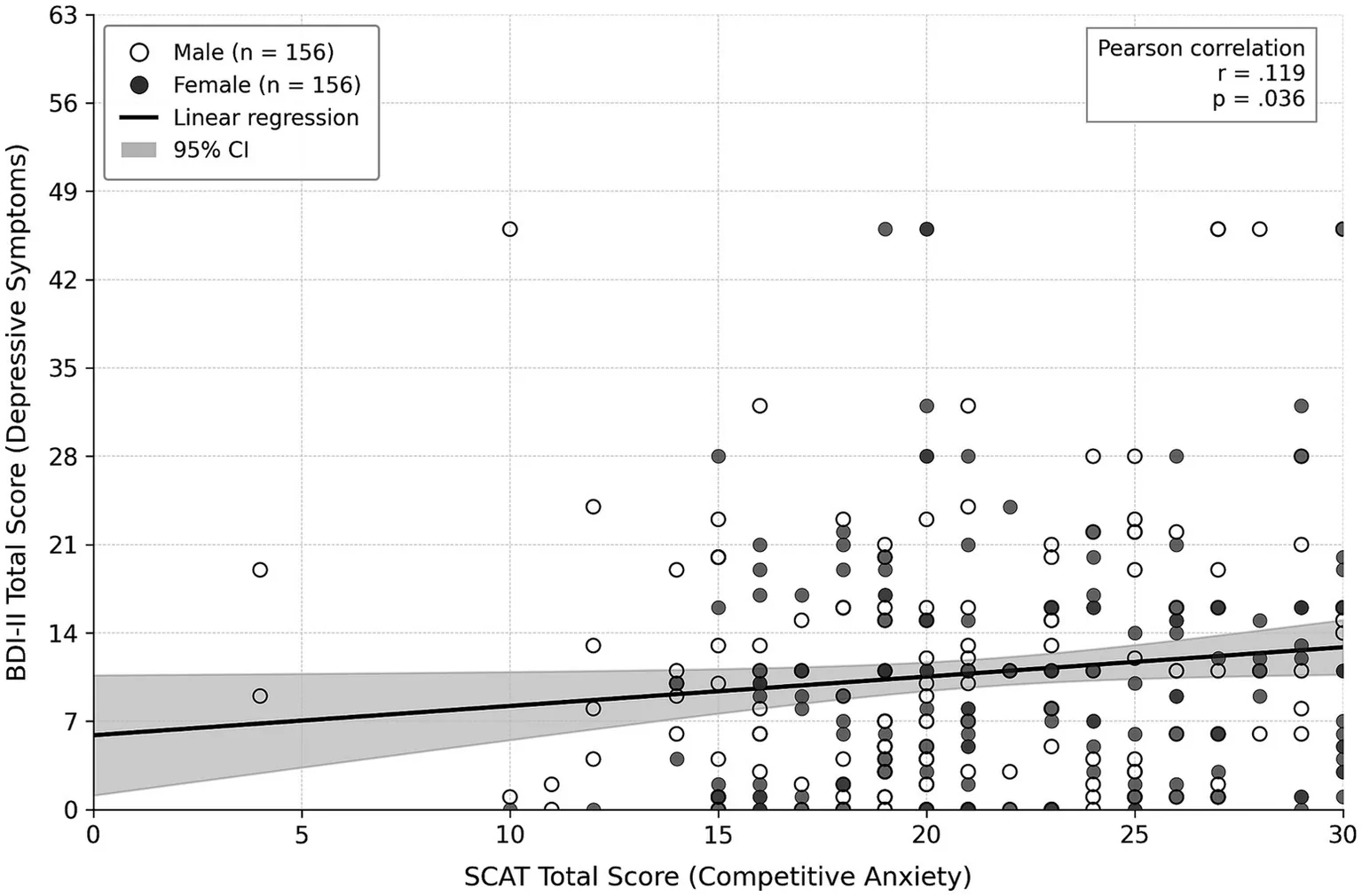
Association between depressive symptom severity and competitive anxiety in competitive tennis players.

Figure 2 illustrates the association between depressive symptoms and competitive anxiety. Correlations between demographic, clinical, and psychological variables and the outcome measures are provided in Supplementary Table S2. Depressive symptom severity demonstrated a significant positive association with competitive anxiety, *r* = 0.119, *p* = 0.036. Exploratory moderation analyses did not identify statistically significant interaction effects for sex, previous diagnosis of depression, or serious injury or illness within the previous 6 months (all interaction *p* values > 0.05).

Higher BDI-II scores were additionally associated with a previous diagnosis of depression, *r* = 0.345, *p* < 0.001, previous suicide attempt, *r* = 0.185, *p* = 0.001, psychotropic medication use, *r* = 0.224, *p* < 0.001, and serious injury or illness within the previous 6 months, *r* = 0.117, *p* = 0.039.

Competitive anxiety demonstrated significant associations with a previous diagnosis of depression, *r* = −0.130, *p* = 0.022, sex, *r* = −0.168, *p* = 0.003, and current location, *r* = −0.144, *p* = 0.011. No significant associations were observed between competitive anxiety and age, tennis experience, psychotropic medication use, or previous suicide attempt.

### Predictors of clinically relevant depressive symptoms

**Figure 3:**
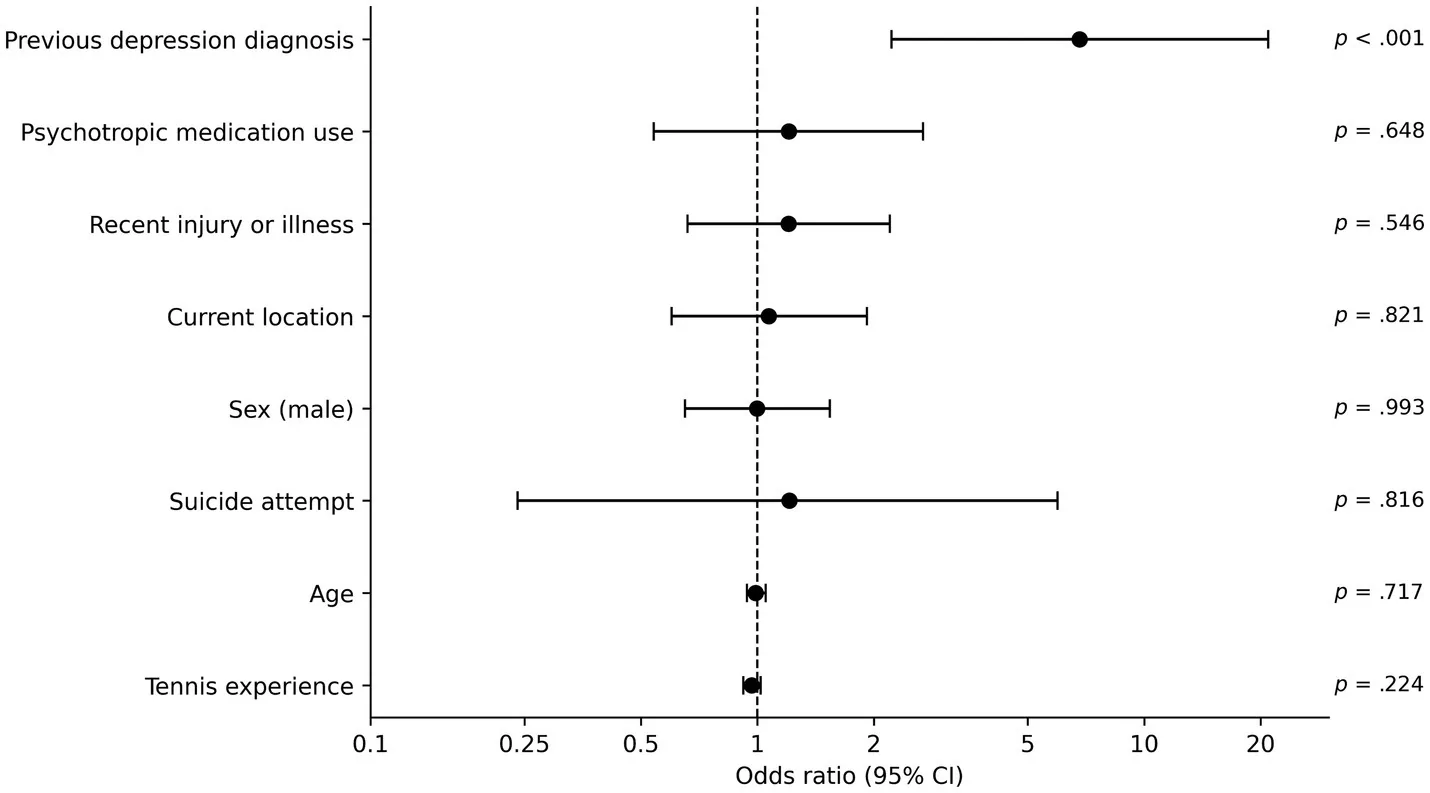
Odds ratios (ORs) with 95% confidence intervals for predictors of clinically relevant depressive symptoms (BDI-II >= 14).

Results of the logistic regression analysis predicting clinically relevant depressive symptoms (BDI-II ≥ 14) are presented in Figure 3, and full model results are provided in Supplementary Table S3. The logistic regression model explained a modest proportion of the variance (Nagelkerke’s *R^2^* = 0.07). A previous diagnosis of depression emerged as the only significant predictor of clinically relevant depressive symptoms, *OR* = 6.81, 95% *CI* [2.22, 20.91], *p* < 0.001. No significant associations were observed for the remaining predictors; however, these findings should be interpreted cautiously because several predictors were infrequent.

### Multivariable predictors of depressive symptom severity and competitive anxiety

**Figure 4:**
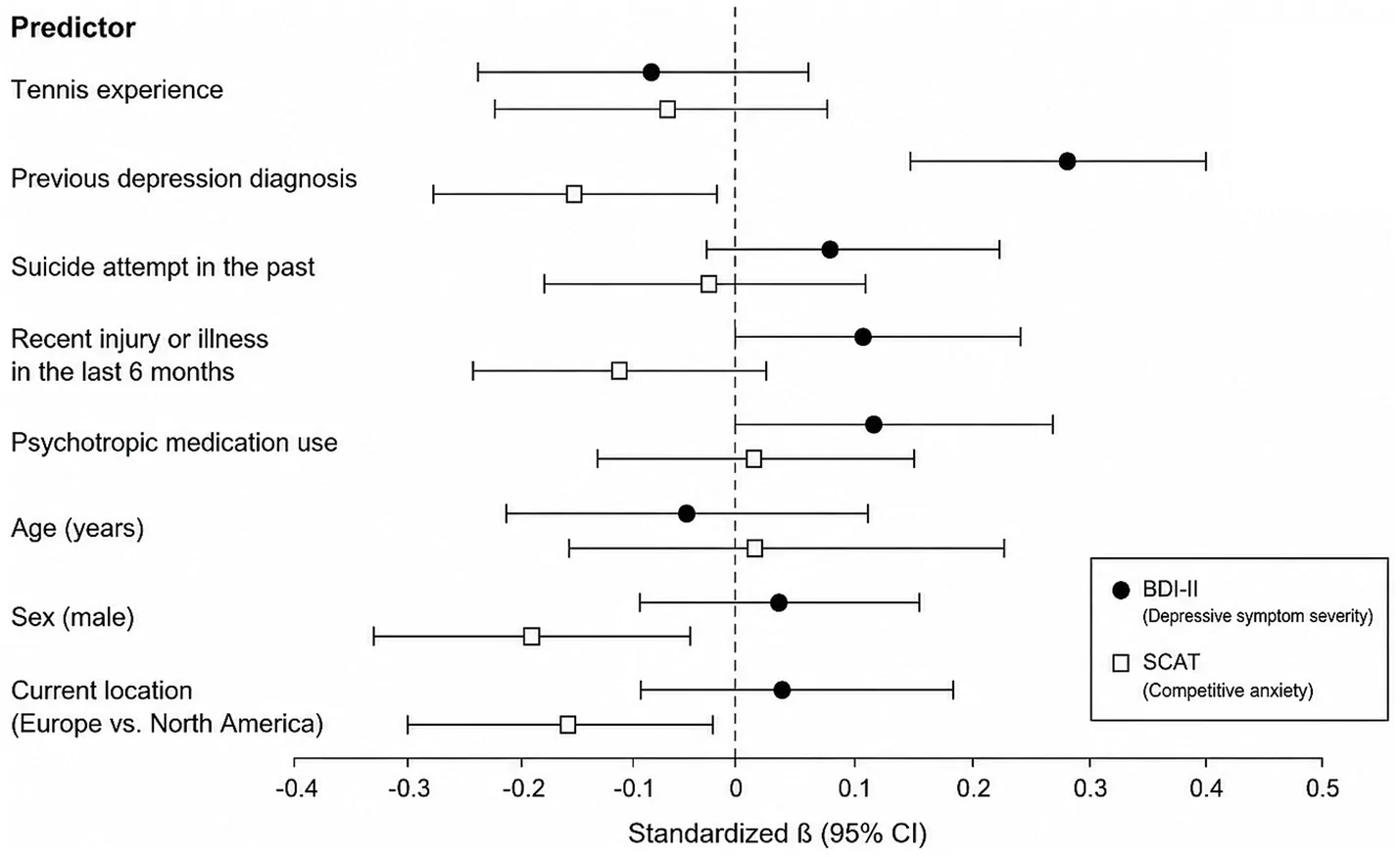
Standardized regression coefficients (*β*) with 95% confidence intervals for predictors of depressive symptoms and competitive anxiety.

Figure 4 summarizes the multivariate linear regression analyses for depressive symptom severity and competitive anxiety, with full model results provided in Supplementary Tables S4, S5. A previous diagnosis of depression (*β* = 0.299, *p* < 0.001), serious injury or illness within the previous 6 months (β = 0.114, *p* = 0.030), and psychotropic medication use (β = 0.122, *p* = 0.033) significantly predicted higher BDI-II scores. No significant associations were observed for tennis experience, previous suicide attempt, age, sex, or current location.

For competitive anxiety, a previous diagnosis of depression (β = −0.130, *p* = 0.031), sex (β = −0.159, *p* = 0.005), and current location (β = −0.143, *p* = 0.010) significantly predicted SCAT scores. Female athletes and athletes located in Europe reported higher competitive anxiety scores. No significant associations were observed for tennis experience, previous suicide attempt, serious injury or illness within the previous 6 months, psychotropic medication use, or age.

## Discussion

Our study investigated depressive symptoms and competitive anxiety in active tennis players and examined demographic and sport-related correlates of psychological distress. The main findings were that one third of the athletes reported clinically relevant depressive symptoms, while competitive anxiety demonstrated a significant positive association with depressive symptom severity. Furthermore, indicators of previous or ongoing psychological burden, including a previous diagnosis of depression, recent injury or illness, and psychotropic medication use, were associated with greater depressive symptom severity. Female athletes additionally reported significantly higher competitive anxiety levels than male athletes. Collectively, these findings highlight the substantial mental health burden in tennis players and suggest that psychological distress in this population may be influenced by both pre-existing vulnerability factors and the specific psychological demands of competitive tennis.

### Prevalence of depressive symptoms and competitive anxiety in tennis players

Clinically relevant depressive symptoms were observed in 33.5% of the investigated tennis players. This finding aligns with growing evidence demonstrating that symptoms of depression and anxiety are common among competitive athletes (1–3). Nevertheless, the prevalence observed in our cohort appears comparatively high relative to several previous investigations in elite athlete populations, where prevalence estimates for depressive symptoms have generally ranged between approximately 19 and 34% (2, 10).

Pluhar et al. (5) reported significantly greater symptoms of anxiety and depression in athletes competing in individual sports compared with team sport athletes. Individualized competitive environments are frequently characterized by increased personal responsibility, repeated performance evaluation, and limited opportunities for emotional regulation during competition, factors previously associated with elevated psychological strain in athletes (3). Within tennis, athletes are continuously exposed to ranking-based competition, prolonged individual performance evaluation, and substantial self-regulatory demands during both training and competition. Frequent travel demands and continuous tournament exposure have been proposed as additional sources of psychological stress in competitive tennis environments.

Competitive anxiety levels were likewise elevated, with 30% of the investigated athletes demonstrating high competitive anxiety scores. A significant positive association between competitive anxiety and depressive symptoms was further observed, supporting previous evidence indicating substantial overlap between anxiety and depressive symptoms in athletic populations (1, 10). Comparable relationships between anxiety symptoms and broader indicators of psychological distress have additionally been described in elite athlete populations (3).

Female athletes demonstrated significantly higher competitive anxiety levels than male players, consistent with epidemiological findings reporting greater anxiety symptom burden among female athletes (2, 21). Differences in stress perception, psychosocial stress exposure, and coping behavior have previously been discussed as potential contributing factors to this increased vulnerability (3). In contrast, depressive symptom severity did not differ significantly between sexes in our cohort, suggesting that sex-related differences may be more pronounced for competitive anxiety than for depressive symptoms within tennis populations.

### Relationship between depressive symptoms and competitive anxiety

A significant but weak positive association between depressive symptoms and competitive anxiety was observed in our cohort. Comparable relationships between anxiety and depressive symptoms have consistently been reported in athletic populations (1, 10) and are similarly well established within broader psychiatric literature (3). Both symptom domains likely reflect overlapping responses to persistent psychological stress within competitive sport environments.

Tennis is characterized by repeated exposure to highly evaluative situations and sustained performance demands, which have been proposed as potential contributors to psychological strain and emotional burden in athletes (3, 22). Although these mechanisms were not examined directly in the present study, they may provide one possible explanation for the observed association.

Within this context, depressive symptoms and competitive anxiety in tennis players appear to represent related but largely distinct dimensions of psychological distress.

### Clinical and sport-related correlates of psychological distress

Indicators of previous or ongoing psychological burden were consistently associated with greater depressive symptom severity in our cohort. In particular, a previous diagnosis of depression emerged as the strongest predictor of depressive symptoms and remained significantly associated with depressive symptom severity in the multivariable analyses. This finding is consistent with existing evidence suggesting that previous mental health problems represent important vulnerability factors for future psychological distress in athletes (2, 3, 23). Depressive symptoms in athletes likely emerge within broader patterns of cumulative psychological vulnerability and chronic stress exposure.

Recent injury or illness was additionally associated with significantly greater depressive symptom severity. Psychological distress following injury has previously been linked to disrupted training continuity, uncertainty regarding return to play, impaired athletic identity, and reduced sport participation (3, 24). Comparable associations between injury burden and depressive symptoms have previously been described in competitive athletes (3, 25).

Psychotropic medication use was likewise associated with higher depressive symptom severity in our cohort. This finding most likely reflects underlying or previously treated mental health conditions rather than medication-related effects themselves. Collectively, these findings suggest that depressive symptoms in tennis players may be more closely associated with indicators of previous psychological burden than with demographic characteristics or the sport-related variables assessed in the present study. Unexpectedly, athletes with a previous diagnosis of depression reported lower competitive anxiety scores. Possible explanations include previous psychological treatment, differences in symptom profiles, reduced exposure to competitive situations, or residual confounding. Given the small number of athletes reporting a previous depression diagnosis, this finding should be interpreted cautiously.

In contrast, neither age nor years of tennis experience demonstrated significant associations with depressive symptoms or competitive anxiety. Geographic location was similarly unrelated to depressive symptom severity, although athletes from Europe reported significantly higher competitive anxiety levels than athletes from North America. The reasons underlying this finding remain unclear but may involve differences in competitive systems, collegiate sport structures, or sociocultural perceptions of athletic performance. Further research is required to better understand potential contextual and environmental influences on psychological distress in tennis players.

### Limitations

Several limitations should be considered when interpreting the findings of our study. First, the cross-sectional study design does not permit conclusions regarding causal relationships between depressive symptoms, competitive anxiety, and the investigated correlates. The observed associations should therefore be interpreted as descriptive rather than causal in nature.

Second, all outcomes were assessed using self-report questionnaires, which may increase the risk of response bias, recall bias, and symptom underreporting or overreporting. In addition, psychological symptoms were not confirmed by structured clinical interviews. The reported prevalence rates therefore reflect symptom burden rather than formal psychiatric diagnoses.

Third, although the study included athletes from different competitive levels and geographic regions, several subgroup sizes remained limited. This may have reduced statistical power for subgroup-specific analyses and restricted the interpretation of regional differences. In addition, the voluntary online recruitment through tennis associations, clubs, and individual athletes may have introduced selection bias, as participation depended on self-selection.

Several predictors included in the regression analyses, such as previous depression diagnosis and previous suicide attempt, occurred infrequently in the present sample. Consequently, the corresponding regression estimates should be interpreted with caution, as low event frequencies may have resulted in unstable estimates and wide confidence intervals.

Furthermore, potentially relevant contextual factors, including training environment, psychosocial support systems, and competition-related stress exposure, were not assessed in detail. The variable assessing serious injury or illness within the previous 6 months combined potentially heterogeneous conditions without further differentiation regarding injury type, illness type, severity, time-loss duration, current status, or whether the condition was tennis-related. In addition, somatic symptoms captured by the BDI-II may have been influenced by recent physical injury or illness.

Moreover, measurement equivalence between the German and English questionnaire versions was not formally examined. Because questionnaire language was closely aligned with geographic region and participants were broadly grouped into Europe and North America rather than analyzed by individual country, the interpretation of geographic comparisons should be made with caution.

The relatively large number of statistical tests performed may have increased the risk of type I error. Accordingly, findings with *p* values close to the significance threshold should be interpreted with caution.

Finally, several potentially relevant tennis-specific variables, including training volume, tournament participation, competitive level, player ranking, travel burden, and financial dependence on tennis, were not assessed. Future longitudinal investigations are therefore warranted to better understand temporal relationships and potential mechanisms underlying psychological distress in competitive tennis players.

## Conclusion

Competitive tennis players demonstrated a substantial burden of depressive symptoms and competitive anxiety, with one third of the investigated athletes reporting clinically relevant depressive symptom severity. Competitive anxiety was significantly but weakly associated with depressive symptoms, supporting a modest relationship between both constructs within competitive sport environments. In addition, previous depression diagnosis, recent injury or illness, and psychotropic medication use were associated with greater depressive symptom severity, highlighting their relevance as clinical correlates in this cohort. These findings highlight the potential relevance of cumulative psychological vulnerability and ongoing stress exposure in this population.

Female athletes additionally reported significantly higher competitive anxiety levels than male athletes, consistent with previous epidemiological findings in elite sport populations. Collectively, these findings support the need for further research evaluating mental health screening and psychological support strategies in competitive tennis. Future longitudinal research is warranted to further clarify temporal relationships and sport-specific mechanisms contributing to psychological distress in this population.

## Data Availability

The raw data supporting the conclusions of this article will be made available by the authors, without undue reservation.
