# Supplementary material for: Prevalence and correlates of depressive symptoms and competitive anxiety in tennis players: a cross-sectional study
Source: Front Public Health. 2026 Aug 26;14:1920866. doi: 10.3389/fpubh.2026.1920866 (PMC13553500; doi:10.3389/fpubh.2026.1920866)
Supplement: Supplementary file 1 [file Data_Sheet_1.PDF]

Supplementary Material

Prevalence and predictors of depressive symptoms and competitive anxiety in tennis players: a cross-sectional study

Table S1. Group differences in depressive symptoms (BDI-II) and competitive anxiety (SCAT) across selected health-related variables.

Table S2. Correlations between demographic, clinical, and psychosocial variables and depressive symptoms (BDI-II) and competitive anxiety (SCAT).

Table S3. Logistic regression analysis predicting clinically relevant depressive symptoms (BDI-II ≥ 14).

Table S4. Multivariate linear regression analysis predicting depressive symptom severity (BDI-II scores).

Table S5. Multivariate linear regression analysis predicting competitive anxiety (SCAT scores).

Table S1. Group differences in depressive symptoms (BDI-II) and competitive anxiety (SCAT) across selected health-related variables

Variable	Type	n	BDI-II mean (SD)	SCAT mean (SD)	*t* Test BDI-II / SCAT
Sex	F	156	10.8 (9.7)	22.2 (4.9)	*t*(310)=-0.11, *p*=.91, *d*=.01
	M	156	10.9 (10.2)	20.5 (5.1)	*t*(310)=3.00, *p*=.003**, *d*=.34
Diagnosed with depression in the past	Yes	20	24.0 (15.8)	18.9 (5.9)	*t*(311)=-6.49, *p*<.001***, *d*=1.50
	No	293	10.0 (9.0)	21.5 (5.0)	*t*(311)=2.30, *p*=.022*, *d*=.53
Suicide attempt in the past	Yes	9	21.6 (19.6)	19.8 (5.9)	*t*(311)=-3.32, *p*=.001***, *d*=1.12
	No	304	10.6 (9.8)	21.4 (5.1)	*t*(311)=0.95, *p*=.341, *d*=.32
Serious injury/illness in the last 6 months	Yes	63	13.2 (11.5)	20.3 (5.2)	*t*(311)=-2.07, *p*=.039*, *d*=.29
	No	250	10.3 (9.6)	21.6 (5.0)	*t*(311)=1.85, *p*=.066, *d*=.26

Note. n = number of participants; BDI-II = Beck Depression Inventory-II; SCAT = Sport Competition Anxiety Test. Significant differences are indicated by asterisks (**p* < .05, ***p* < .01, ****p* < .001).

Table S2. Correlations between demographic, clinical, and psychosocial variables and depressive symptoms (BDI-II) and competitive anxiety (SCAT)

Variable (reference)	Correlation with BDI-II	Correlation with SCAT
Tennis experience	*r*=-.097, *p*=.085	*r*=-.015, *p*=.778
Diagnosed with depression in the past	*r*=.345, *p*<.001***	*r*=-.13, *p*=.022*
Suicide attempt in the past	*r*=.185, *p*=.001***	*r*=-.054, *p*=.341
Serious injury/illness in the last 6 months	*r*=.117, *p*=.039*	*r*=-.104, *p*=.066
Use of psychotropic medication	*r*=.224, *p*<.001***	*r*=-.016, *p*=.779
Age in years	*r*=-.088, *p*=.119	*r*=.031, *p*=.583
Sex	*r*=.006, *p*=.914	*r*=-.168, *p*=.003**
Current location (Europe vs. North America)	*r*=.008, *p*=.890	*r*=-.144, *p*=.011*

Note. BDI-II: Beck Depression Inventory-II; SCAT: Sport Competition Anxiety Test. Significant differences are indicated by asterisks (**p* < .05, ***p* < .01, ****p* < .001).

Table S3. Logistic regression analysis predicting clinically relevant depressive symptoms (BDI-II ≥ 14)

Variable (reference)	OR	95% CI	*z*-value	*p*-value
Tennis experience	.967	0.92–1.02	-1.215	.224
Diagnosed with depression in the past	6.811	2.22–20.91	3.352	<.001***
Suicide attempt in the past	1.209	0.24–5.97	.233	.816
Serious injury or illness in the last 6 months	1.204	0.66–2.20	.603	.546
Use of psychotropic medication	1.205	0.54–2.68	.457	.648
Age in years	.990	0.94–1.05	-.361	.717
Sex (male)	.998	0.65–1.54	-.009	.993
Current location (Europe vs. North America)	1.070	0.60–1.92	.226	.821

Note. OR = odds ratio. Significant differences are indicated by asterisks (**p* < .05, ***p* < .01, ****p* < .001).

Table S4. Multivariate linear regression analysis predicting depressive symptom severity (BDI-II scores)

**Variable (reference)**	***β***	**95% CI**	***p*-value**
Tennis experience	-.078	[-0.23, 0.06]	.291
Diagnosed with depression in the past	.299	[0.18, 0.40]	<.001***
Suicide attempt in the past	.099	[-0.01, 0.21]	.066
Serious injury/illness in the last 6 months	.114	[0.01, 0.22]	.030*
Use of psychotropic medication	.122	[0.01, 0.24]	.033*
Age in years	-.040	[-0.18, 0.12]	.590
Sex	.045	[-0.07, 0.14]	.406
Current location (Europe vs. North America)	.045	[-0.07, 0.07]	.400

Note. Significant differences are indicated by asterisks (*$p < .05$, **$p < .01$, ***$p < .001$).

Table S5. Multivariate linear regression analysis predicting competitive anxiety (SCAT scores)

**Variable (reference)**	***β***	**95% CI**	***p*-value**
Tennis experience	-.076	[-0.23, 0.08]	.330
Diagnosed with depression in the past	-.130	[-0.25, -0.02]	.031*
Suicide attempt in the past	-.015	[-0.13, 0.10]	.787
Serious injury/illness in the last 6 months	-.092	[-0.20, 0.02]	.098
Use of psychotropic medication	.021	[-0.10, 0.14]	.726
Age in years	.025	[-0.12, 0.19]	.747
Sex	-.159	[-0.28, -0.05]	.005**
Current location (Europe vs. North America)	-.143	[-0.25, -0.03]	.010*

Note. Significant differences are indicated by asterisks (*$p < .05$, **$p < .01$, ***$p < .001$).
